# Formation and Regulation Mechanism of Ascorbic Acid in Sweet Pepper and Chili Pepper at Different Growth Stages

**DOI:** 10.3390/foods14213675

**Published:** 2025-10-28

**Authors:** Zhe Zhang, Xinxin Li, Hongxiao Zhang, Zhanghong Yu, Yanqin Fan, Yaning Meng, Libin Yan

**Affiliations:** 1 Institute of Cash Crops, Hebei Academy of Agriculture and Forestry Sciences, Shijiazhuang 050051, China; luanzhi914@163.com (Z.Z.); 13837253988@163.com (X.L.); zhang185287@126.com (H.Z.); yzhong2021@163.com (Z.Y.); nkyfanyq@126.com (Y.F.); 2 College of Horticultural Science & Technology, Hebei Normal University of Science & Technology, Qinhuangdao 066600, China; 3 Hebei Province Engineering Research Center for Vegetables, Shijiazhuang 050051, China

**Keywords:** pepper, ascorbic acid, transcriptome, metabolome, differentially expressed genes, APX3

## Abstract

To investigate the molecular mechanisms underlying ascorbic acid (AsA) accumulation in pepper fruits and to identify key genes involved in its biosynthesis, we performed integrated metabolomic and transcriptomic analyses on two pepper cultivars—T41 (sweet pepper) and 22-5 (chili pepper)—at three developmental stages, including young fruit, green ripe, and color change stages. The results show that AsA content in both cultivars was significantly higher at the green ripe stage than the young fruit stage, with T41 exhibiting significantly higher AsA levels than 22-5 at both the young fruit and green ripe stages. Transcriptomic analysis identified a total of 24,433 differentially expressed genes (DEGs). KEGG pathway enrichment analysis revealed that genes associated with AsA biosynthesis were mainly enriched in the “ascorbate and aldarate metabolism” pathway. Follow-up validation confirmed APX3 as the most likely candidate gene responsible for the difference in AsA content between the two pepper fruit types, with its expression pattern negatively correlating with AsA accumulation. This study unveils the molecular regulatory mechanisms underlying AsA biosynthesis and provides a theoretical foundation for breeding pepper cultivars with elevated AsA levels. This study provides valuable insights into the molecular regulation of AsA biosynthesis and lays a theoretical foundation for breeding high-AsA pepper varieties.

## 1. Introduction

Pepper is an important economic crop, valued not only as a seasoning for human meals but also for its rich nutrient contents, such as carotenoids, flavonoids, ascorbic acid, capsaicin, and various mineral elements [1]. The ascorbic acid (Vitamin C) content in the fruits of pepper is relatively high, with some types even surpassing that of kiwifruit, making it an important vegetable in daily life [2]. Since some animals (including humans) lack the ability to synthesize ascorbic acid, they must obtain it from external sources [3]. Plants are the main source of ascorbic acid. Ascorbic acid in plants not only has antioxidant properties, reducing free radicals in plants and enhancing their stress resistance, but also participates in plant growth and development, such as cell division, cell wall metabolism, cell expansion, shoot apical meristem formation, root development, photosynthesis, flowering regulation, and leaf senescence regulation. Thus, ascorbic acid could serve as an essential nutrient for healthy plant growth [4]. AsA is a core metabolite indispensable for healthy plant growth, elucidating the biosynthetic pathway and regulatory mechanisms of AsA, which have significant theoretical and practical value for improving crop stress resistance and nutritional quality.

Currently, four AsA synthesis pathways have been identified in plants, including the L-galactose pathway, D-galacturonic acid pathway, myo-inositol pathway, and L-gulose pathway [5]. Among these, the L-galactose pathway is recognized as the main pathway for AsA synthesis in plants [6]. The L-galactose pathway starts with D-mannose-6-phosphate and, through a series of catalytic reactions by key enzymes such as GME, GPP, GGP, and GDH, ultimately generates AsA. In the AsA metabolic process, AsA can be oxidized to monodehydroascorbate (MDHA) by enzymes, such as ascorbate peroxidase (APX), ascorbate oxidase (AO), and monodehydroascorbate reductase (MDHAR). MDHA can further spontaneously convert to dehydroascorbate (DHA). DHA can be reduced back to AsA by dehydroascorbate reductase (DHAR), thus maintaining redox homeostasis in plants [4]. Studies in crops such as strawberry [7], kiwifruit [8,9], and tomato [10,11] have found that GME is the main rate-limiting gene for AsA synthesis, while in peppers, the GGP gene has been identified as the main differential gene for AsA synthesis [12]. It is worth noting that although APX, AO, and MDHAR all have the ability to break down ascorbic acid, they have some functional differences. MDHAR only unidirectionally reduces ascorbic acid, while APX and AO, which reduce ascorbic acid, also decompose excess hydrogen peroxide in the body, maintaining redox balance in plants [13]. Current research suggests that ascorbate peroxidase (APX) and ascorbate oxidase (AO) genes also play an important role in regulating the AsA content in plants [14,15]. In tomato, RNA interference to suppress the expression of the AO gene can significantly reduce AO enzyme activity, thereby increasing the AsA content in the fruit [16]. The expression of ascorbate peroxidase (CsAPX1) in tomato leaves and its activity are significantly regulated by light/dark conditions, with AsA accumulation and APX activity being inhibited in the dark [17]. Transgenic plants with increased CsAPX1 expression exhibit smaller reductions in AsA levels and APX activity during postharvest storage than wild-type plants [18]. Similarly, in sweet pepper, the expression of the APX gene significantly increases during fruit ripening. However, as the fruit matures, the activity of APX diminishes, resulting in a reduction of AsA content [19]. Moreover, high expression of the AO gene in various plants has been found to be associated with reduced AsA content, as AO oxidizes AsA to generate dehydroascorbate [20]. These research findings indicate that regulating the expression of APX and AO genes can effectively adjust the AsA content in plants, which is significant for enhancing the antioxidant capacity and nutritional value of crops.

Although the AsA metabolic pathway has been clarified, differences still exist in the synthesis patterns among different crops and even among different varieties of the same crop. In this study, the synthesis mechanisms of AsA in sweet peppers and line peppers were analyzed and compared through metabolomics and transcriptomics, with the aim of screening out the key genes regulating AsA synthesis and ultimately providing a theoretical basis for quality breeding.

## 2. Materials and Methods

### 2.1. Experimental Materials

The peppers used in the experiment were germplasm resources collected, identified, and selected by researchers from the Hebei Academy of Agricultural and Forestry Sciences. T41 is a sweet pepper and 22-5 is a chili pepper. The peppers were sown on 8 January 2023, using 72-hole plug trays for seedling cultivation. They were transplanted to a plastic greenhouse in the Modern Agricultural Experimental Garden of the Hebei Academy of Agricultural and Forestry Sciences on 27 March. The growth environment and management level were consistent.

**Figure 1:**
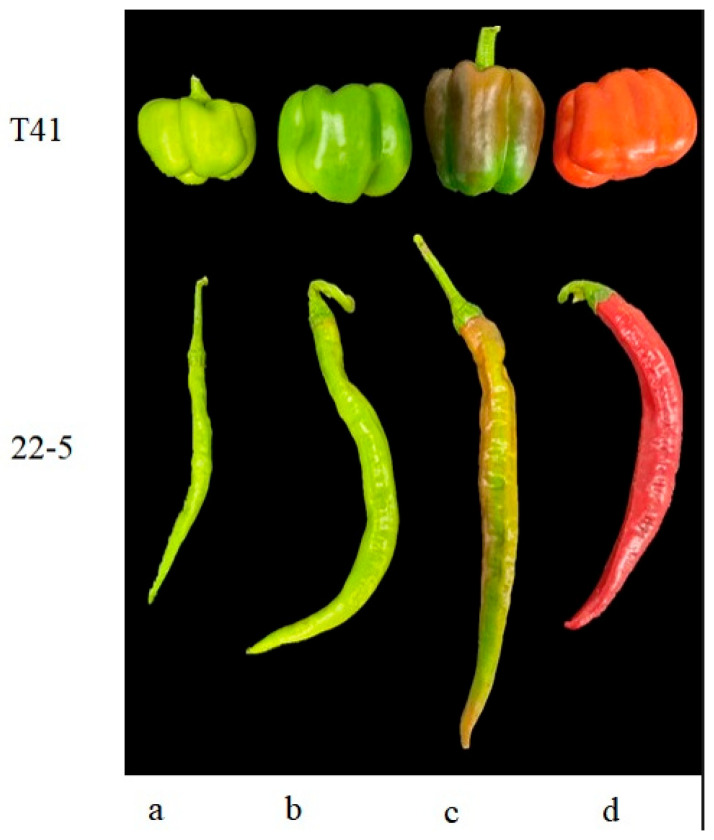
Sweet peppers and chili peppers at different growth stages. (**a**) Young fruit stage; (**b**) mature green stage; (**c**) breaking color stage; (**d**) mature stage.

Six normally growing plants with consistent growth vigor were selected for each variety, and fruits were harvested at the young fruit stage, green ripe stage, and color change stage (Figure 1). Two pepper fruits at the same node were selected from each plant. The fruits of the two plants were mixed into one sample, with three biological replicates at each period. Immediately after AsA quantification, the residual tissue fragments were plunged into liquid nitrogen for 5 min and transferred to a −80 °C ultrafreezer to preserve metabolite integrity prior to metabolomics, RNA sequencing, and qRT-PCR validation.

### 2.2. Sample Preparation and Extraction

Biological material was first lyophilized in a Scientz-100F vacuum freeze-dryer (Ningbo Scientz Biotechnology Co. LTD., Ningbo, China), then the sample was pulverized for 1.5 min at 30 Hz in an MM 400 mixer mill with one zirconia bead. Fifty milligrams of the resulting powder were taken up in 1.2 mL 70% methanol and vortex-mixed for 30 s at 30 min intervals (six cycles in total). Following centrifugation (12,000 rpm, 3 min), the supernatant was passed through a 0.22-μm SCAA-104 filter (ANPEL, Shanghai, China) prior to UPLC-MS/MS injection [21].

### 2.3. UPLC Separation

Extracts were resolved on an ExionLC™ AD system (AB Sciex Pte. Ltd., Framingham, MA, USA) coupled to an Agilent SB-C18 column (Agilent Technologies (China) Co., Ltd., Beijing, China) (100 mm × 2.1 mm, 1.8 µm). Eluent A was water containing 0.1% formic acid; eluent B was acetonitrile with 0.1% formic acid. The gradient rose from 5% B to 95% B over 0–9 min, held at 95% B for 1 min, returned to 5% B in 0.1 min and re-equilibrated for 2.9 min. The flow rate was 0.35 mL min^−1^, the column temperature was 40 °C, and the injection volume was 4 µL. The column effluent was diverted on-line to a 4500 QTRAP mass spectrometer through a high-pressure switching valve [22].

### 2.4. ESI-QTRAP-MS/MS

A ESI source operated in polarity-switching mode supplied the following settings: probe temperature of 550 °C; ion spray voltage of (IS) ±5.5/−4.5 kV; ion source gas I (GSI) at 50 psi, gas II (GSII) at 60 psi, curtain gas (CUR) at 25 psi; collision-activated dissociation (CAD) set to “high”. MRM transitions were acquired with nitrogen as collision gas (medium). The declustering potential and collision energy were optimized per precursor–product ion pair; transition lists were scheduled in 1 min windows to match metabolite retention times [23].

### 2.5. RNA Extraction and Illumina Sequencing

Total RNA was isolated from frozen pericarp (modified CTAB protocol [24]) for three biological replicates of each pepper accession. mRNA was reverse-transcribed and converted to indexed cDNA libraries verified on an Agilent 2100 Bioanalyzer (Agilent Technologies (China) Co., Ltd., Beijing, China). Libraries were pooled and sequenced on an Illumina HiSeq 4000 (Illumina Inc., San Diego, CA, USA). Adapters, reads containing ≥10% ambiguous bases, and QPhred ≤ 20 positions covering >50% of the read were discarded. Clean reads were aligned to reference genome (NCBI) (https://www.ncbi.nlm.nih.gov/genome/?Term=Capsicum%20annuum, accessed on 16 September 2024) using HISAT2. Gene-level counts were normalized to FPKM [25]. Differential expression genes were determined with DESeq2 |(log2(Fold Change)| > 1 and padj < 0.05) [26] and functionally annotated via GO and KEGG enrichment [27].

### 2.6. qRT-PCR Validation

Primer Premier 5.0 software (Premier Biosoft International, Palo Alto, CA, USA) designed gene-specific primers for ten DEGs. Cycling conditions were 95 °C for 5 min, followed by 40 cycles of 95 °C for 15 s and 60 °C for 30 s. Each biological sample was run in triplicate, and relative expression was normalized to Actin (GenBank: GQ339766).

## 3. Results

### 3.1. Principal Component Analysis of Overall Samples

**Figure 2:**
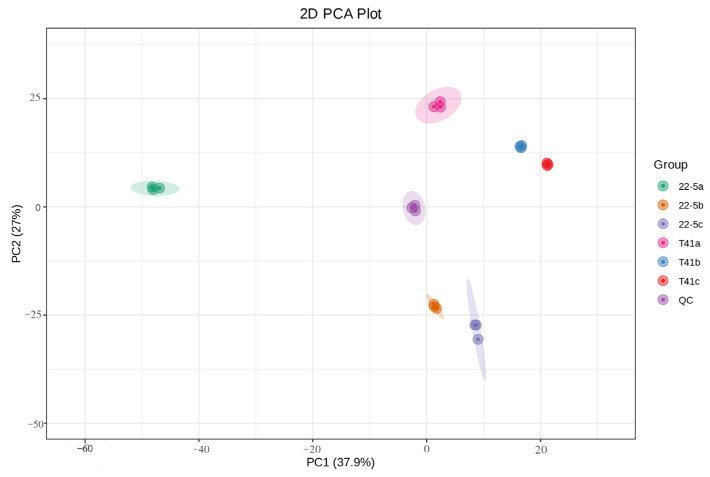
PCA analysis of the total sample.

**Figure 3:**
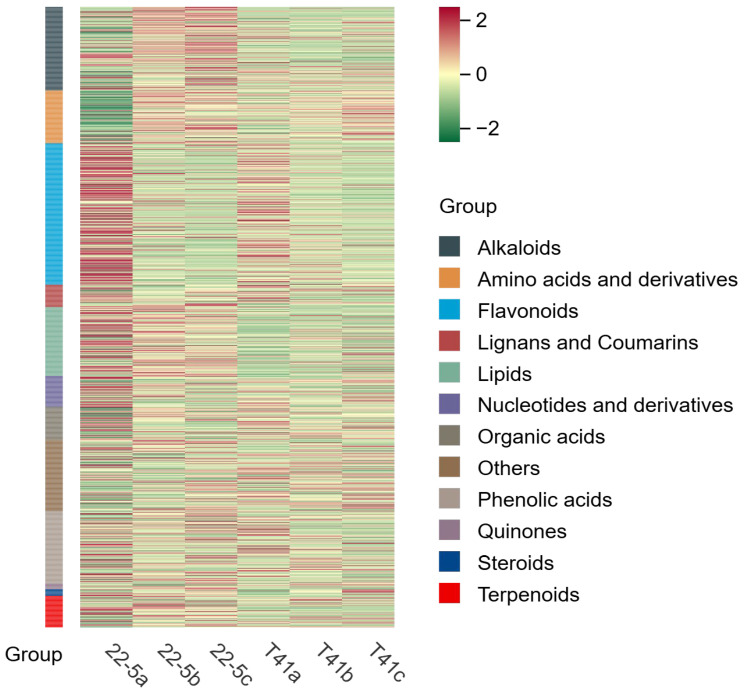
Sample cluster analysis diagram.

Principal component analysis was conducted on all experimental samples, revealing that with PC1 at 37.9% and PC2 at 27%, distinct varieties and developmental stages were clearly distinguishable (Figure 2). At the same time, the same samples did not show significant separation, and the three biological replicates clustered closely together, which is in line with biological variation characteristics. These results generally indicate that the experiment produced consistent, stable, and trustworthy results. Furthermore, a total of 1223 metabolites were identified by ultra-high-performance liquid chromatography–tandem mass spectrometry (UPLC-MS/MS) coupled to an in-house spectral library. These metabolites were taxonomically classified into the following chemical superfamilies: flavonoids, alkaloids, phenolic acids, lipids, amino acids and their derivatives, organic acids, terpenoids, nucleotides and their derivatives, lignans and coumarins, steroids, quinones, and a residual “others” category (Figure 3 and Table S1).

### 3.2. Analysis of Differentially Expressed Metabolites in Different Pepper Cultivars

**Figure 4:**
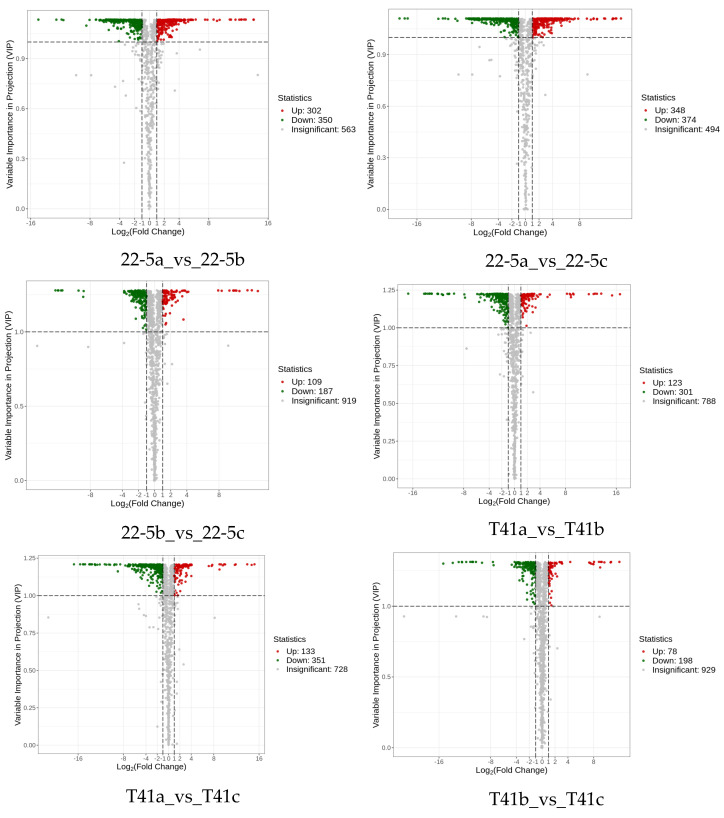

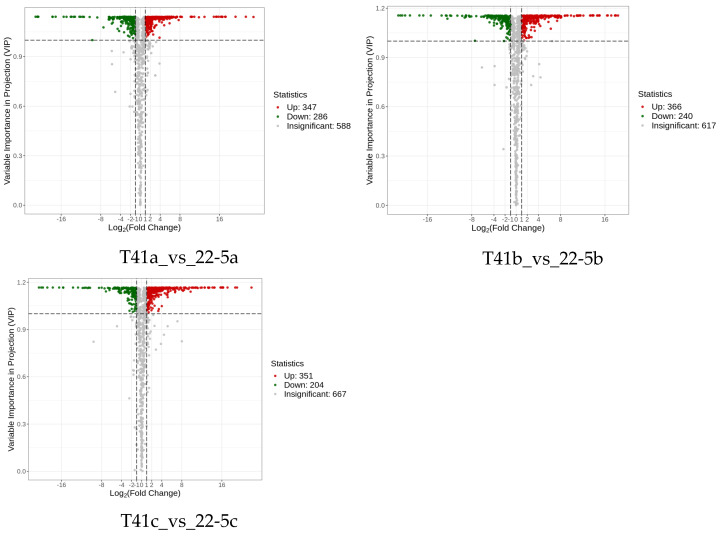
MA diagram of differential expressed metabolites. Each dot in the MA plot represents a single metabolite: green dots indicate down-regulated differential metabolites, red dots indicate up-regulated differential metabolites, and gray dots represent metabolites that were detected but not significantly different. The x-axis shows the log_2_ fold-change (log_2_FC) of the relative abundance of a metabolite between the two groups (vertical dashed lines at |log_2_FC| > 1); the larger the absolute value, the greater the difference in relative abundance. The y-axis denotes the VIP value (horizontal dashed line at VIP > 1.0); the higher the value, the more significant and reliable the metabolite is considered to be.

Subsequently, differentially expressed metabolite analysis was performed on the two varieties of peppers at different developmental stages. As we found, a total of 4648 DEMs were identified in the 22-5 variety. Compared with 22-5a, 302 metabolites were up-regulated and 350 were down-regulated in 22-5b. In the 22-5a vs. 22-5c comparison group, 348 metabolites were up-regulated and 374 were down-regulated in 22-5c. In the 22-5b vs. 22-5c comparison group, a total of 296 DEMs were identified, among which 109 were up-regulated and 187 were down-regulated in 22-5c. In the T41 variety, compared with T41b, 123 metabolites were up-regulated and 301 were down-regulated in T41a. In the T41a vs. 22-5a comparison group, 347 metabolites were up-regulated and 286 were down-regulated in 22-5a. In the T41b vs. 22-5b comparison group, 626 DEMs were identified, among which 366 were up-regulated and 260 were down-regulated in 22-5b. In the T41c vs. 22-5c comparison group, 351 metabolites were up-regulated and 204 were down-regulated in 22-5c (Figure 4).

**Figure 5:**
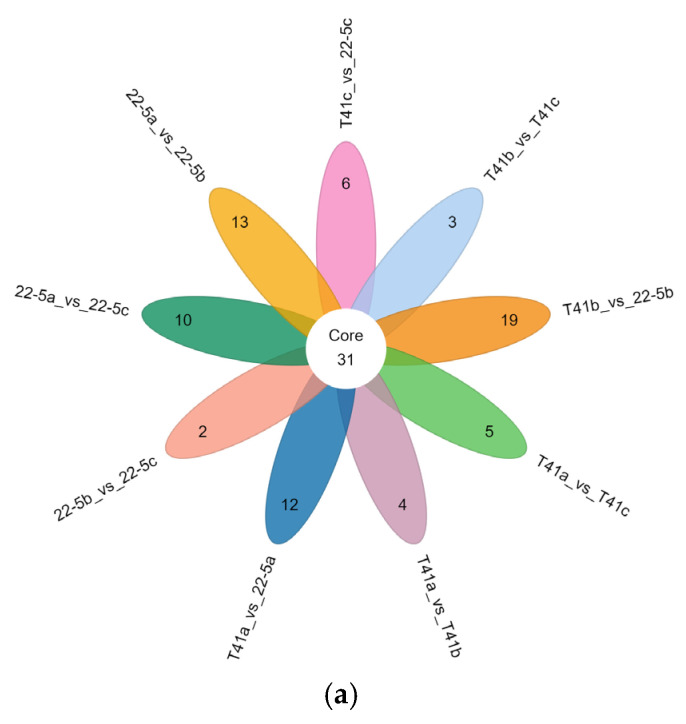

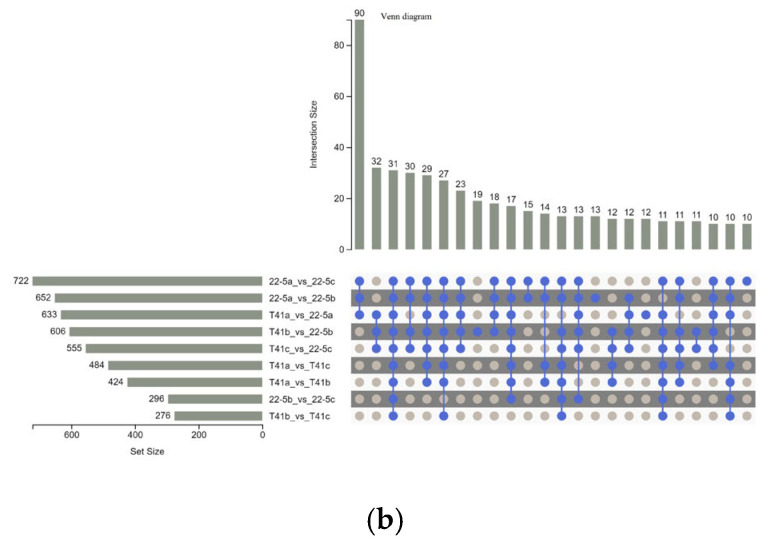
Venn diagrams of differential expressed metabolites in each group. (**a**) petal Venn diagram. Each circle in the diagram represents one comparison group; the numbers in the overlapping regions indicate how many differential metabolites are shared between groups, whereas the numbers in the non-overlapping regions indicate group-specific differential metabolites. Panel (**b**) is an UpSet plot: the blue dots and connecting lines denote the numbers of metabolites common to the corresponding comparison groups. Each gray dot represents one group.

Furthermore, Venn diagram analysis revealed that 31 common DEMs were shared across the nine groups (Figure 5a). Additionally, there were 90 common DEMs among three groups, including 22-5a vs. 22-5b, 22-5a vs. 22-5c, and T41a vs. 22-5a, while other groups shared 10 to 32 DEMs (Figure 5b).

The set size in Figure 5b is a statistical measure of the number of metabolites present in each set. The intersection size on the right is the statistical result of the number of metabolites after taking the intersection of various different sets. The single point below represents an element unique to a certain group, and the line connecting the points represents the intersection unique to different groups.

### 3.3. KEGG Enrichment Analysis of DEMs in the Two Varieties

**Figure 6:**
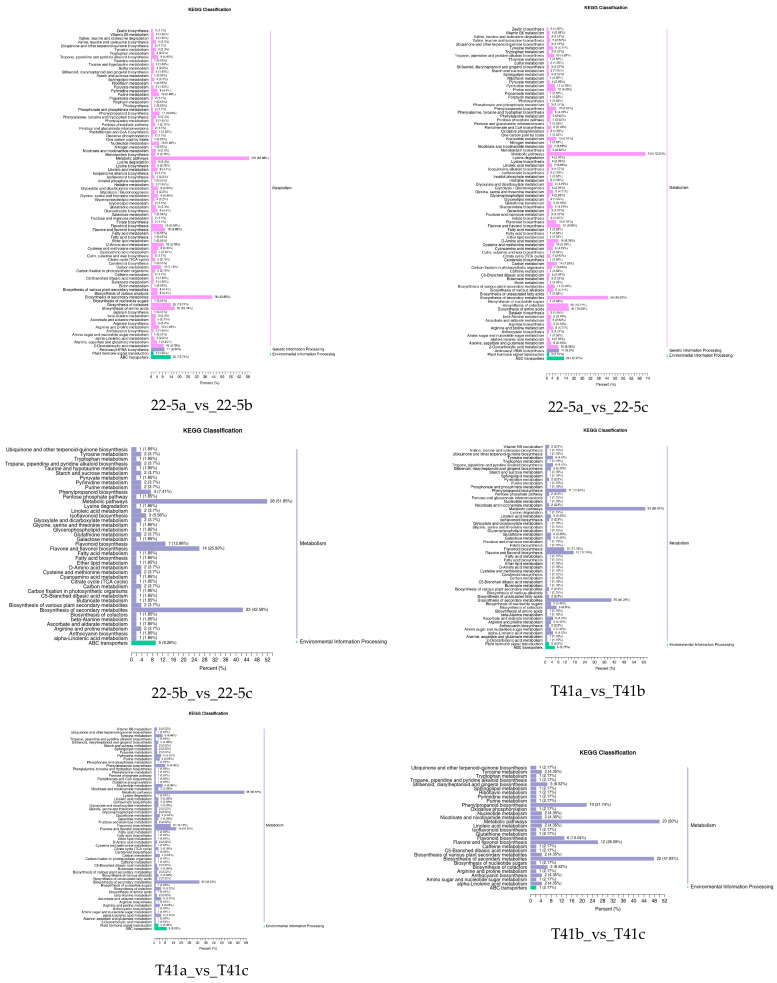

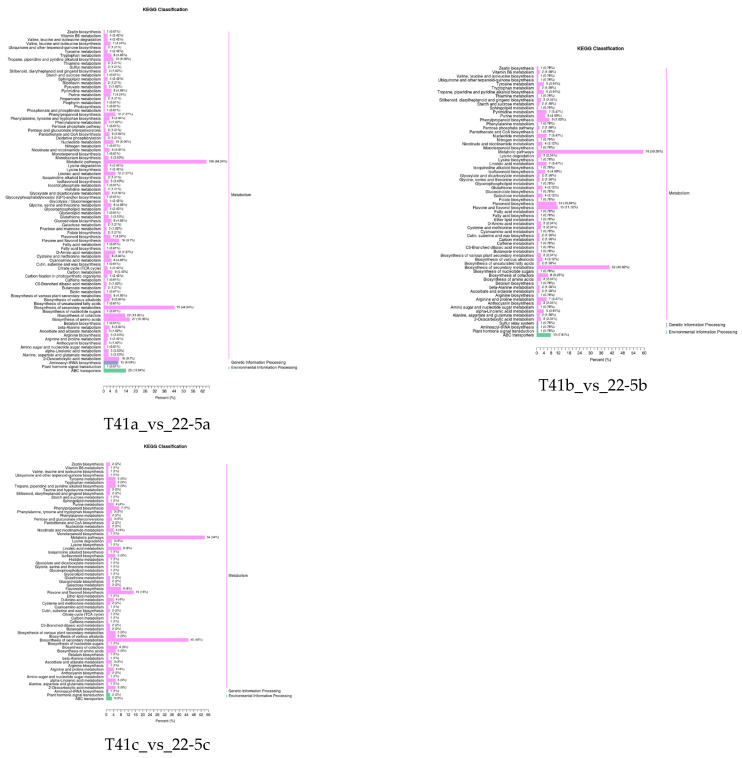
KEGG enrichment classification diagram of differential expressed metabolites.

Further, the DEMs from the two varieties were further subjected to KEGG functional enrichment analysis. The results indicate that in the 22-5 variety, both 22-5a_vs_22-5b and 22-5a_vs_22-5c had 88 significantly enriched pathways. In the 22-5b_vs_22-5c comparison group, DEMs were enriched in 42 pathways. In the T41 variety, DEMs were enriched in 56 pathways in the T41a_vs_T41b comparison group, 61 pathways in T41a_vs_T41c, 30 pathways in T41b_vs_T41c, 87 metabolic pathways in T41a_vs_22-5a, 68 metabolic pathways in T41b_vs_22-5b, and 65 metabolic pathways in T41c_vs_22-5c. Among these, five pathways related to vitamin C biosynthesis metabolism were identified, namely metabolic pathways (ko01100), biosynthesis of secondary metabolites (ko01110), ascorbate and aldarate metabolism (ko00053), glutathione metabolism (ko00480), and biosynthesis of cofactors (ko01240). Among the five pathways, ascorbate and aldarate metabolism is the main synthetic metabolic pathway for ascorbic acid, with five DEMs identified. The five DEMs were mainly concentrated in comparison groups 22-5a_vs_22-5b, 22-5a_vs_22-5c, T41a_vs_22-5a, T41a_vs_T41b, T41a_vs_T41c, and T41b_vs_22-5b (Figure 6).

### 3.4. Comparison of AsA Content in Fruits of T41 and 22-5 at Different Developmental Stages

**Figure 7:**
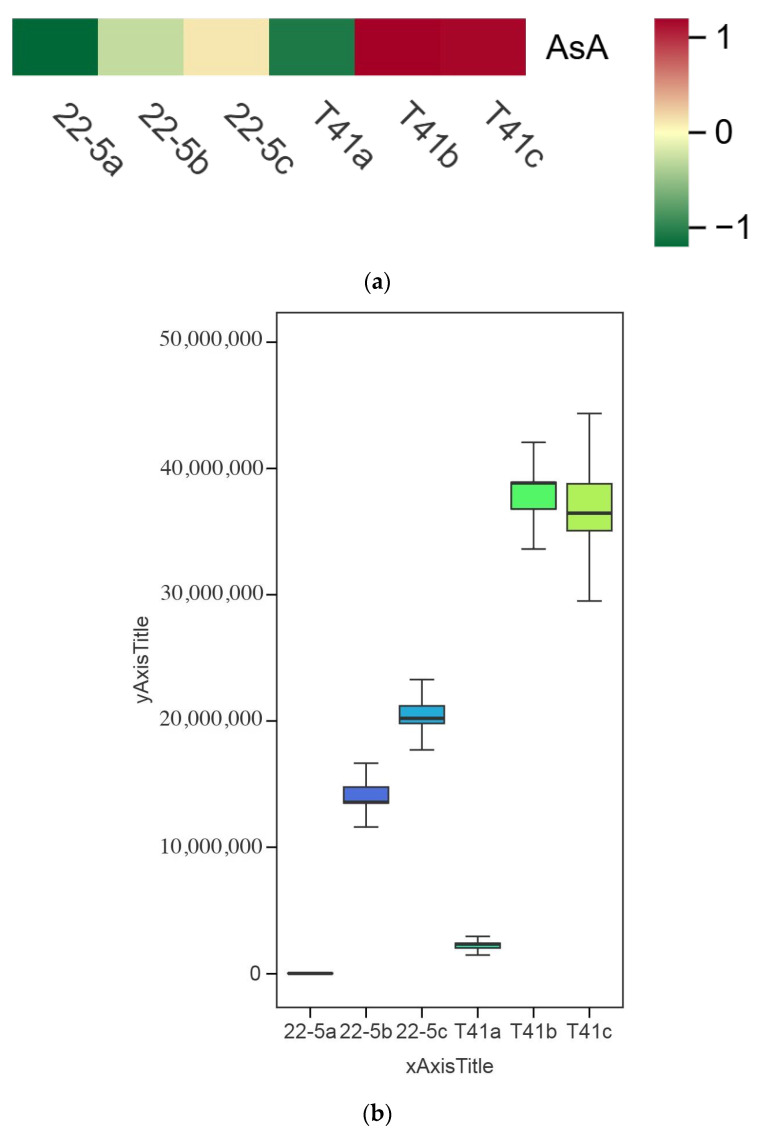

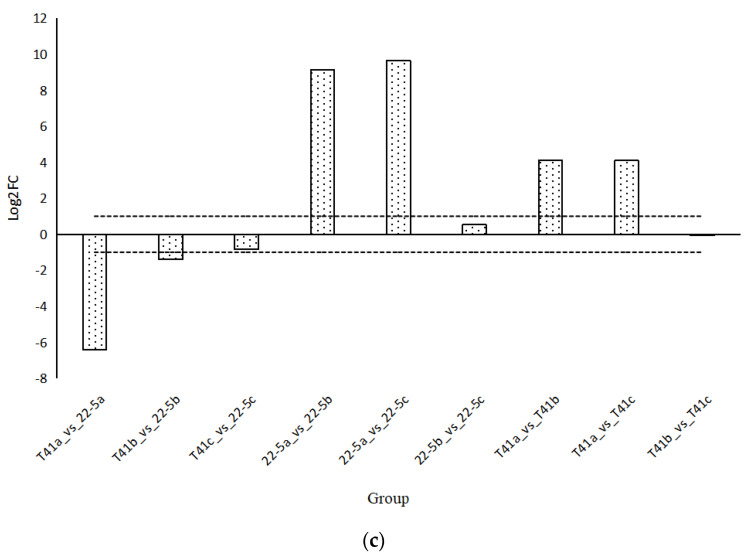
Comparison of AsA relative content in sweet peppers and chili peppers at different growth stages. (**a**) Heat map of relative ascorbic acid (AsA) content in pepper fruit at different developmental stages. (**b**) Analysis of variance (ANOVA) for relative AsA content in pepper fruit at different developmental stages. (**c**) Fold-change analysis of relative AsA content in each comparison group. In panel (**c**), the horizontal dashed line indicates the threshold of |log_2_FC| = 1.

Changes in the AsA content in the fruits of T41 and 22-5 were investigated at different developmental stages. The results show that the AsA content in the pericarp of T41 and 22-5 was significantly higher at the green ripe stage than at the young fruit stage (Figure 7). In the color change stage, the AsA content in the pericarp of both T41 and 22-5 was significantly higher than that at the young fruit stage, with no significant difference observed compared to the green ripe stage. Further comparison of the AsA content in the pericarp of the two varieties in the same period shows that the AsA content in the fruits of T41 was significantly higher than that of 22-5 at both the young fruit stage and the green ripe stage. However, during the color change period, there was no significant difference in AsA content between the pericarp of T41 and 22-5. Subsequently, we only selected the fruits at the young fruit stage and the green-ripe stage of T41 and 22-5 for comparative analysis of the transcriptome.

### 3.5. Transcriptome Data Analysis of the Two Varieties

To better identify the molecular mechanisms regulating vitamin C synthesis, we performed transcriptome sequencing on fruits of 22-5 and T41 at different developmental stages. A total of 159.62 Gb of clean data was obtained, with Q20 ≥ 97.89%, Q30 ≥ 93.81%, and the GC content was between 41% and 43%. The alignment efficiency of reads from the two varieties with the reference genome was between 94.50% and 95.51%, which indicates that the data utilization rate fell within the expected range, indicating that the chosen reference genome was appropriate for further analysis.

### 3.6. Differential Expressed Gene Analysis of Young Fruit and Green Ripe Stages in the Two Varieties

**Figure 8:**
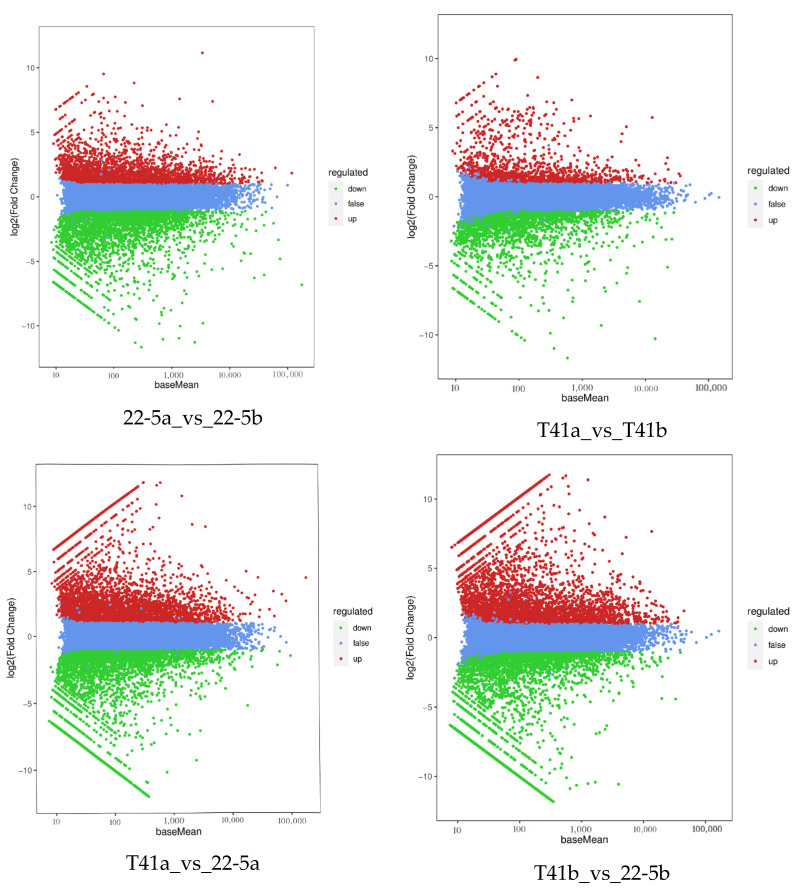
MA diagram of differentially expressed genes.

**Figure 9:**
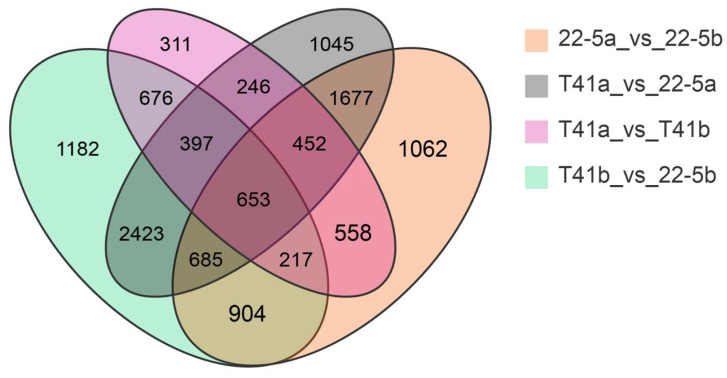
Venn diagram of differential expressed genes.

Transcriptomic analysis was concentrated on the young fruit and green ripe stages, as the differences in pericarp AsA content between the two types were most pronounced during these phases. The 24,433 DEGs were identified in the four comparison groups of T41a_vs_22-5a, T41b_vs_22-5b, 22-5a_vs_22-5b, and T41a_vs_T41b. In the 22-5a vs. 22-5b comparison group, 2662 genes were up-regulated and 3546 genes were down-regulated in 22-5b. In T41a vs. 22-5a, 3845 genes were up-regulated and 3733 genes were down-regulated in 22-5a. In T41a vs. T41b, 1323 genes were up-regulated and 2187 genes were down-regulated in the T41b group. Transcriptomic comparison between T41b and 22-5b revealed 7137 DEGs, with 3745 up-regulated and 3392 down-regulated in line 22-5b (Figure 8). Further analysis of these DEGs using Venn diagrams identified 904 common candidates (Figure 9).

### 3.7. KEGG Enrichment Analysis of Differentially Expressed Genes in Young Fruit and Green Ripe Stages of the Two Varieties

**Figure 10:**
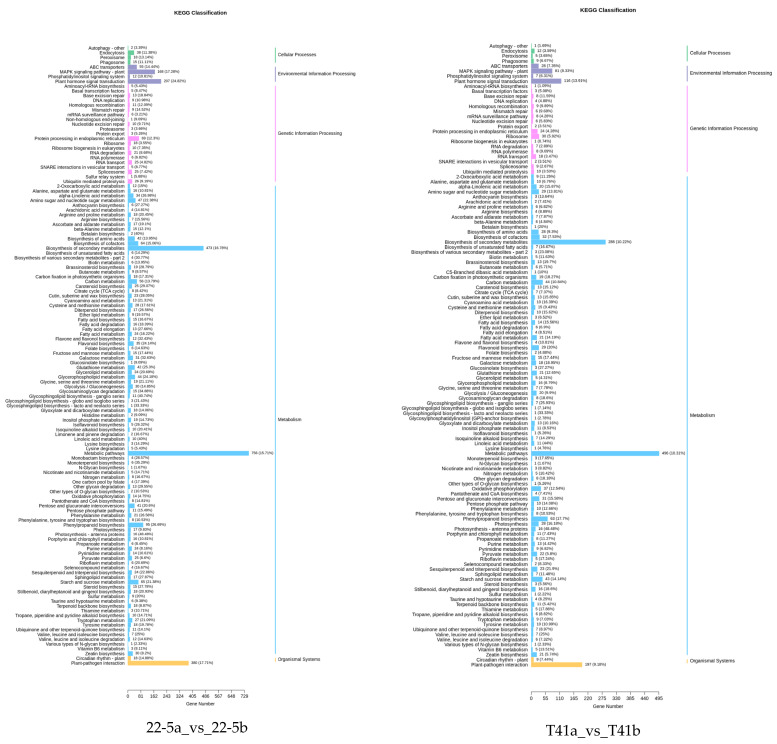

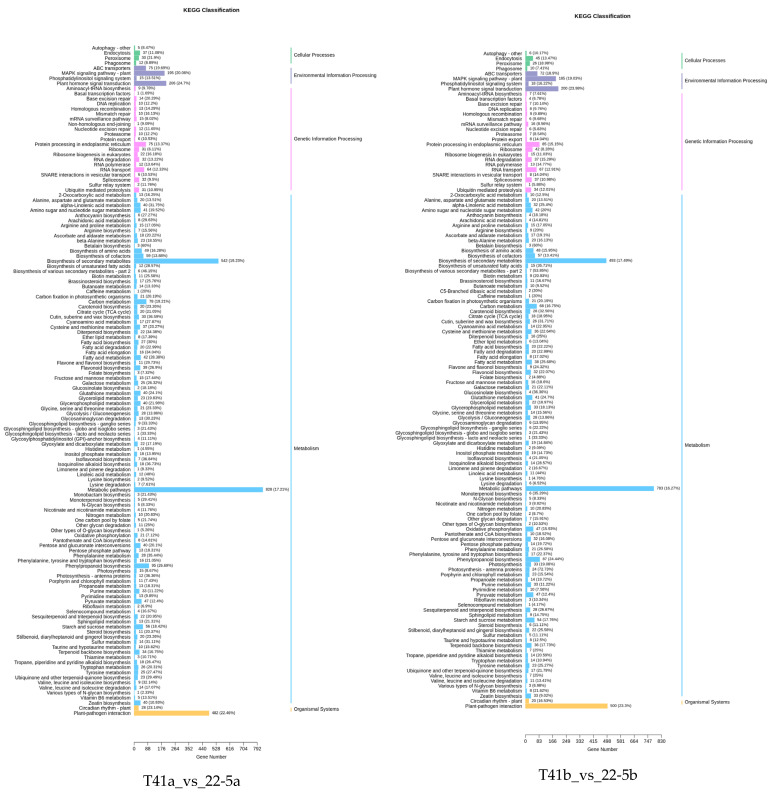
Classification Diagram of KEGG enrichment of differentially expressed genes.

KEGG enrichment analysis was performed on the differentially expressed genes in the 22-5a_vs_22-5b, T41a_vs_T41b, T41a_vs_22-5a, and T41b_vs_22-5b comparison groups. The results show that there were 131, 125, 133, and 131 significantly enriched pathways, respectively. Among these, there were five pathways related to vitamin C biosynthesis metabolism, namely metabolic pathways (ko01100), biosynthesis of secondary metabolites (ko01110), ascorbate and aldarate metabolism (ko00053), glutathione metabolism (ko00480), and biosynthesis of cofactors (ko01240). Among the five pathways, ascorbate and aldarate metabolism is the main anabolic pathway for ascorbic acid. Eighteen DEGs were identified in the ascorbate and aldarate metabolism pathway, mainly in comparison groups 22-5a_vs_22-5b and T41a_vs_22-5a (Figure 10).

### 3.8. WGCNA-Based Co-Expression Network Analysis Across Growth Stages

In this study, Weighted Gene Co-Expression Network Analysis (WGCNA) was employed to explore gene interconnections and construct co-expression networks. These networks were established based on the expression patterns of genes across all sampled tissues, utilizing pairwise correlation coefficients. Highly correlated genes, indicating strong co-expression relationships, were clustered into distinct modules.

**Figure 11:**
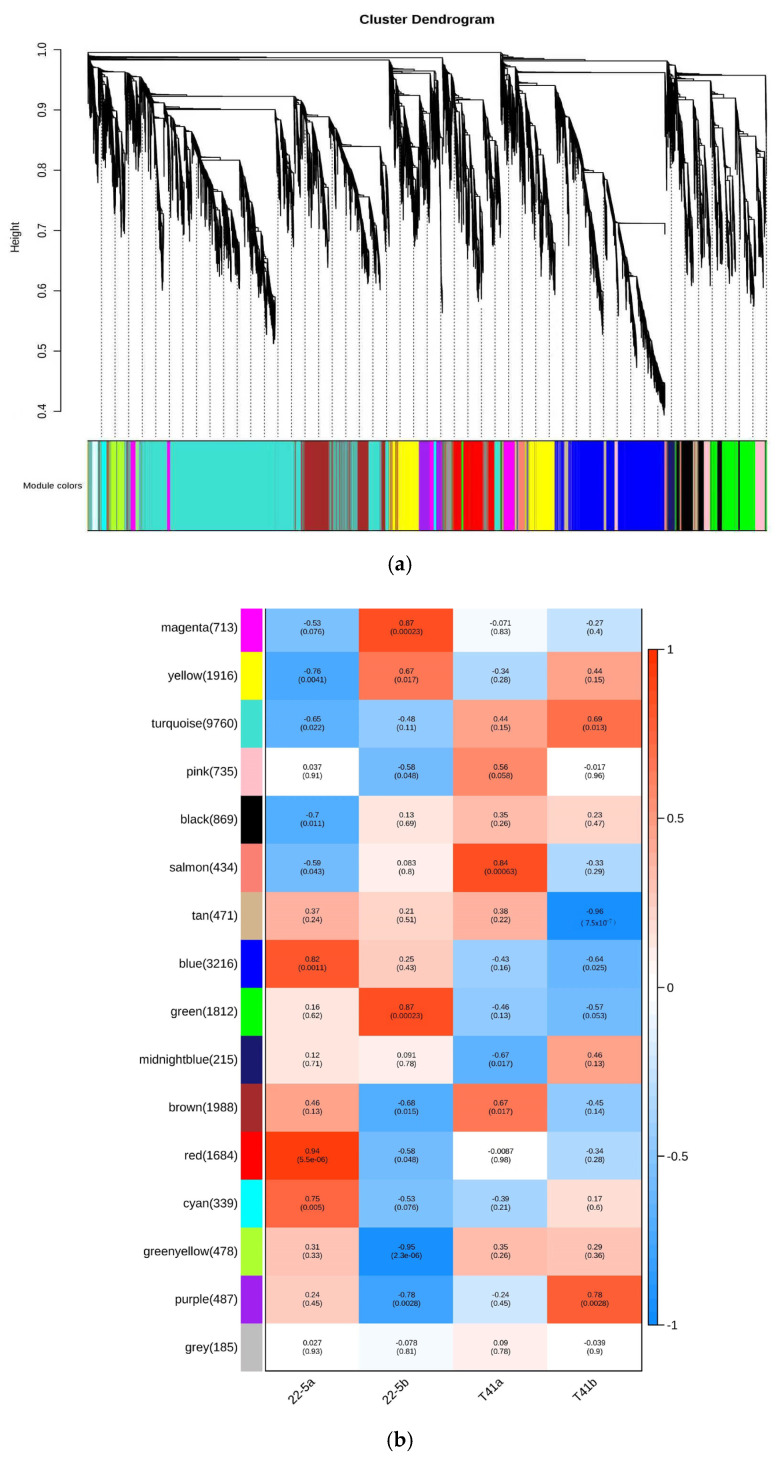
Co-expression network analysis plot. (**a**) Co-expression network dendrogram; (**b**) module chart of co-expression network analysis.

As illustrated in the dendrogram (Figure 11a), a total of 16 modules were identified, each represented by a unique color and composed of genes with similar expression trends. The expression pattern of each module was summarized by its eigengene, which captures the dominant expression profile of the module. These eigengenes exhibited distinct associations with specific tissue types, reflecting tissue-specific gene expression.

Notably, the red module, comprising 1684 genes, showed pronounced accumulation specifically in sample 22-5a. Similarly, the green module, with 1812 genes, was predominantly expressed in 22-5b. The salmon module, containing 434 genes, exhibited specific enrichment in T41a, while the purple module, including 487 genes, was notably expressed in T41b (Figure 11b).

### 3.9. Analysis of Gene Expression Related to AsA Synthesis in Peppers

**Figure 12:**
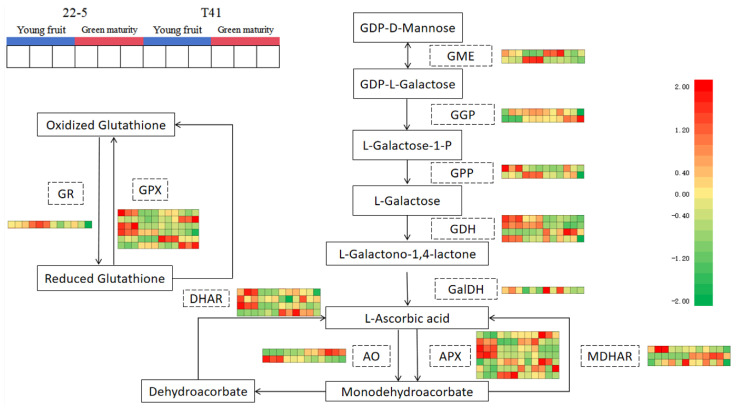
Ascorbate biosynthesis and recycling pathway.

Subsequently, 34 genes involved in the synthesis and metabolism of AsA in pepper fruits were identified among the 11 pathway genes in the ascorbate and aldarate metabolism pathway. Further analysis of the transcriptome data from the young fruit and green ripe stages identified 16 DEMs (Figure 12).

Compared with the T41a group, five genes were significantly up-regulated (including one *AO* gene, two *APX* genes, and two *GPX* genes in the 22-5a group), and a total of five genes were significantly down-regulated (including one *GGP* gene, one *DHAR* gene, one *AO* gene, one *MDHAR* gene, and one *GPX* gene) in the 22-5a group. Compared with the T41b group, the 22-5b group showed a significant increase in the expression of four genes, namely one *GME* gene, one *AO* gene, and two *APX* genes. A total of three genes were significantly down-regulated, including one *AO* gene, one *MDHAR* gene, and one *GPX* gene. In the T41a vs. T41b comparison group, four genes were significantly down-regulated, namely one AO gene, two APX genes, and one GPX gene. In the 22-5a_vs_22-5b comparison group, nine genes were differentially expressed, among which only one GGP gene was up-regulated, while eight genes were down-regulated, including one DHAR gene, one AO gene, three APX genes, and three GPX genes (Table 1).

Furthermore, correlation analysis was conducted between the identified genes and their metabolites related to AsA. The results show that LOC107875751, LOC107851279, and LOC107875737 were significantly positively correlated with AsA content, while LOC107875512 and LOC107859857 showed significant negative correlation (Table 1).

Meanwhile, compared with 22-5a, LOC107875751 was significantly up-regulated, and LOC107875512, LOC107859857, LOC107848404, LOC107842932, LOC107859857, LOC107859858, LOC107875254, LOC107854033, and LOC107848492 were significantly down-regulated in the 22-5b group. In the T41a vs. T41b group, LOC107875254, LOC107848492, LOC107875512, LOC107859857, and LOC107859858 were significantly down-regulated. In the T41a vs. 22-5a group, LOC107854033, LOC107859858, LOC107875512, LOC107848492, LOC107840121, and LOC107859857 were significantly up-regulated, while LOC10785254, LOC107851279, LOC10785751, LOC107864479, LOC107873361, and LOC107848561 were significantly down-regulated.

**Figure 13:**
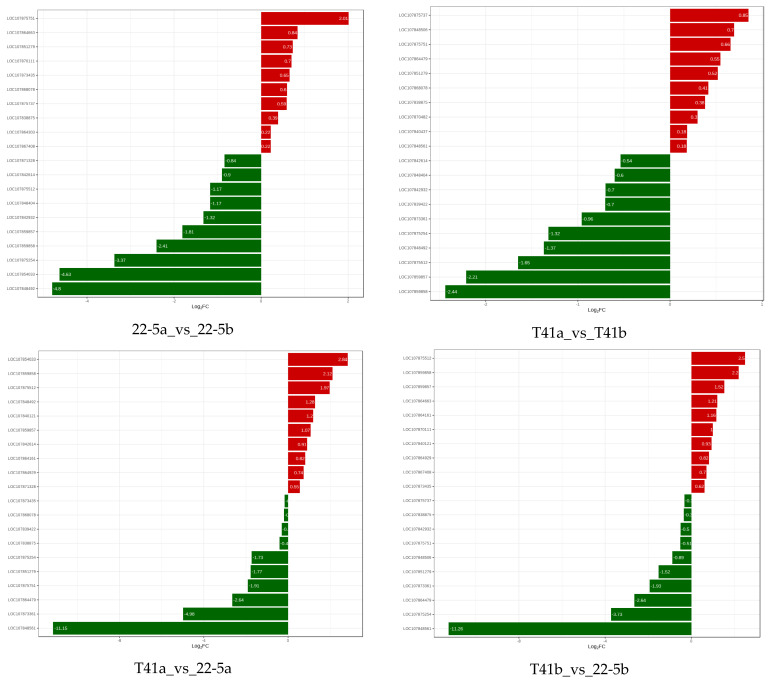
Relative expression levels of pepper genes among different groups.

In the T41b_vs_22-5b group, LOC107875512, LOC107859858, LOC107859857, LOC107864663, LOC107864161, and LOC10780111 were significantly up-regulated, while LOC107851279, LOC107873361, LOC107864479, LOC10785254, and LOC107848561 were significantly down-regulated. Finally, two genes, LOC107875512 and LOC107859857, were obtained and translated into AO and APX, respectively (Figure 13).

### 3.10. Differential Expression of APX3 and AO Genes at Different Developmental Stages of Chili Peppers by qRT-PCR

**Figure 14:**
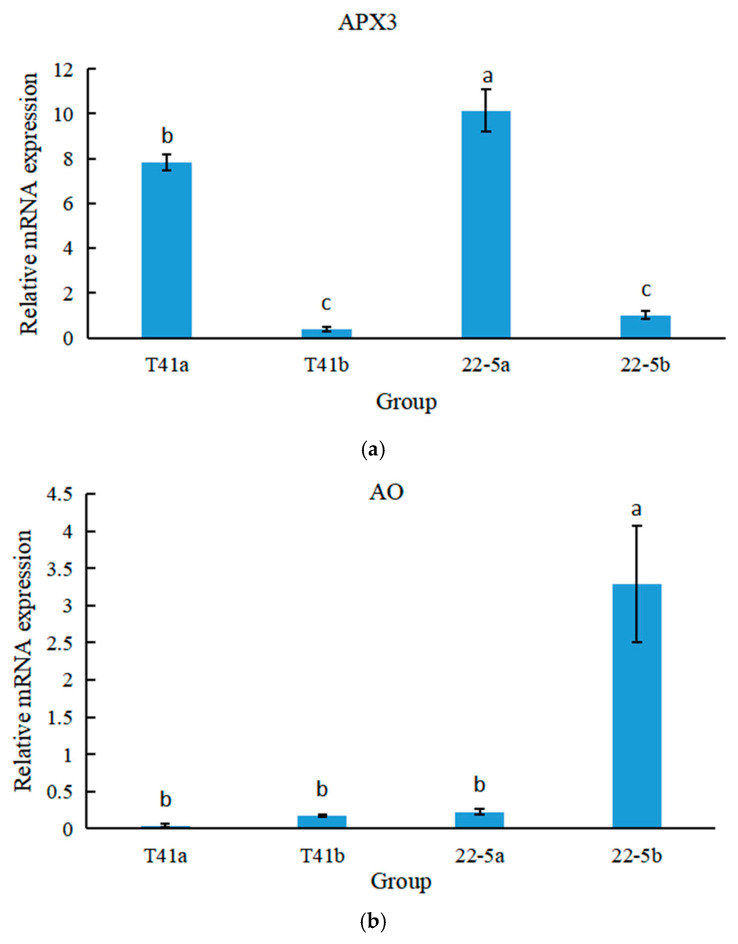
The relative expression levels of Figure 12 APX (**a**) and AO (**b**) genes at different growth stages of peppers. Different lowercase letters indicate significant differences among groups (*p* < 0.05).

qRT-PCR was used to analyze the expression specificity of the APX3 and AO genes at different growth and development stages of peppers. The results show that in both T41 and 22-5 varieties of peppers, the expression level of APX3 was significantly higher at the young fruit stage than at the green ripe stage (Figure 14a). Meanwhile, the expression level of APX in T41 was lower than that in 22-5 during both young fruit and the green ripe stages, with a significant difference at the young fruit stage. As shown in Figure 14b, in both T41 and 22-5 varieties of peppers, only the expression level of AO in the green ripe stage of 22-5 was significantly higher than that at the young fruit stage, with no significant differences among other groups. Therefore, only APX3 showed an opposite trend to AsA content in both transcriptome and qRT-PCR, which may be the main gene responsible for the differences in AsA content between the two types of chili pepper fruits.

## 4. Discussion

Ascorbic acid (vitamin C) is an essential vitamin for humans [28]. As humans are incapable of endogenous AsA synthesis, obtaining this essential nutrient through dietary sources is necessary. Sweet peppers and chili peppers, as important vegetable crops in daily diets, have a high AsA content and are significant dietary sources of vitamin C [29]. Studies have shown that the AsA content in chili pepper fruits dynamically changes with the progression of growth. The AsA content in chili pepper fruits begins to accumulate at the young fruit stage and basically reaches a peak at the green ripe stage, with significant variation among different varieties. For example, Chiaiese et al. found that the AsA accumulation in the PEP10 variety decreased after the green ripe stage, while the AsA content in the PEP1 variety increased rapidly after the color change stage and reached the highest value at the mature stage [30]. Our study found that both tested chili pepper types exhibited a similar AsA accumulation pattern: accumulation began at the young fruit stage, stabilized at the green ripe stage, and no significant increase in AsA content occurred after the color change stage. This result is in line with some previous studies, further confirming the significant variety specificity of AsA accumulation in chili peppers. Therefore, we suggest that future research should systematically classify and study different germplasm resources to deeply explore the genetic regulatory mechanisms of AsA accumulation in chili peppers.

Currently, four AsA biosynthesis pathways have been identified in plants, among which the L-galactose pathway is recognized as the main synthetic pathway [31]. Yuan et al. confirmed that L-galactose dehydrogenase (GDH) in Chinese cabbage plays a key regulatory role in AsA synthesis [32]. In this study, transcriptome data across diverse chili pepper varieties demonstrated a significant enrichment of AsA synthesis-related genes in the ascorbate and aldarate metabolism pathway. In this pathway, we compared the fruit of sweet pepper line T41 and chili pepper line 22-5 at the young fruit and green mature stages and identified two differentially expressed genes: LOC107875512 (AO) and LOC107859857 (APX3). Further comparative analyses of the transcriptome data for the two pepper lines at different developmental stages, together with qRT-PCR validation, showed that, among all the AsA-metabolism-related genes examined, only APX3 exhibited a significant negative correlation between its expression pattern and the AsA-accumulation dynamics of the two cultivars. Transcriptome profiling of mango fruit development by Hassam Tahir et al. [33] also revealed that APX plays a pivotal role in regulating AsA levels in fruit. Similarly, silencing the mitochondrial APX gene in tomato fruit significantly increased vitamin C (AsA) accumulation, largely because the down-regulation of APX reduced the rate of AsA oxidation, thereby promoting its retention [16]. In the present study, APX3 expression declined markedly as pepper fruit developed, a trend that aligns with the previous findings in tomato. We therefore propose that APX3 is the major candidate gene underlying the difference in AsA accumulation between the two pepper lines.

Although ascorbate oxidase (AO) is traditionally viewed as a “consumption factor” of AsA within the plant AsA–metabolic network, an increasing body of association and transgenic evidence indicates that AO transcript abundance is only weakly, if at all, correlated with final AsA content in fruit [34]. Consistently, our qRT-PCR results reveal no coherent relationship between AO transcript levels and the observed changes in AsA content.

The APX (ascorbate peroxidase) gene family plays a pivotal role in plant ascorbic acid (AsA) metabolism by catalyzing the oxidation of AsA to dehydroascorbate (DHA) while reducing H_2_O_2_ to H_2_O and O_2_. This reaction not only scavenges reactive oxygen species (ROS), alleviating oxidative damage, but also markedly enhances plant stress tolerance [35,36]. Transgenic evidence shows that over-expression of cytosolic APX1 in Arabidopsis lowers ion leakage by 42% and increases the maximal photochemical efficiency of photosystem II (Fv/Fm) by 25% under methyl viologen (MV)-induced oxidative stress; conversely, the apx1/apx2 double mutant accumulates 3.8-fold more H_2_O_2_ and exhibits a 65% reduction in survival after 6 h at 38 °C, demonstrating that the APX family functions as a core “safety valve” in maintaining cellular redox homeostasis and protecting plants from oxidative stress [37]. Based on these data, we hypothesize that the pepper cultivars with high APX expression in our study may possess superior antioxidant capacity and stress tolerance; however, this assumption remains to be tested in follow-up experiments.

## 5. Conclusions

In this study, an integrated metabolomic and transcriptomic approach was employed to dissect the molecular mechanisms underlying ascorbic acid (AsA) accumulation in pepper fruit. Comparative transcriptome profiling of different cultivars revealed that genes involved in AsA biosynthesis are predominantly enriched in the “ascorbate and aldarate metabolism” pathway. Subsequent validation identified APX3 as the most likely candidate gene responsible for the observed difference in fruit AsA content between the two pepper types. These findings not only clarify the molecular regulatory network of AsA metabolism in pepper fruit but also provide a theoretical framework for the systematic classification of future germplasm resources. Further work should focus on elucidating how APX3 influences AsA levels and on evaluating its contribution to plant stress tolerance, laying the groundwork for quality-oriented breeding programs.

## Figures and Tables

**Table 1:** Correlation analysis between AsA content and transcriptome.

Gene ID	Correlation Analysis			
	22-5a_vs_22-5b	T41a_vs_T41b	T41a_vs_22-5a	T41b_vs_22-5b
LOC107875751	1.00 **	0.88 *	0.98 **	0.88 **
LOC107851279	0.98 **	0.84 *	0.98 **	0.95 **
LOC107875512	−0.97 **	−0.84 *	−0.94 *	−0.98 *
LOC107859857	−0.98 **	−0.94 **	−0.89 *	−0.96 *
LOC107875737	0.99 **	0.96 **	0.82 *	0.91 *

Note: * *p* < 0.05, ** *p* < 0.01.

## Data Availability

The original contributions presented in this study are included in the article/Supplementary Materials. Further inquiries can be directed to the corresponding authors.

## Supplementary Materials

The following supporting information can be downloaded at https://www.mdpi.com/article/10.3390/foods14213675/s1: Table S1: All sample data.

## References

[1] Antonio A.S., Wiedemann L.S.M., Junior V.V. (2018). The genus Capsicum: A phytochemical review of bioactive secondary metabolites. RSC Adv..

[2] Palma J.M., Terán F., Contreras-Ruiz A., Rodríguez-Ruiz M., Corpas F.J. (2020). Antioxidant Profile of Pepper (*Capsicum annuum* L.) Fruits Containing Diverse Levels of Capsaicinoids. Antioxidants.

[3] Navarre D.A., Zhu M., Hellmann H. (2022). Plant Antioxidants Affect Human and Gut Health, and Their Biosynthesis Is Influenced by Environment and Reactive Oxygen Species. Oxygen.

[4] Skrovankova S., Mlcek J.J.H. (2025). Antioxidant Potential and Its Changes Caused by Various Factors in Lesser-Known Medicinal and Aromatic Plants. Horticulturae.

[5] Ntagkas N., Woltering E.J., Marcelis L.F. (2018). Light regulates ascorbate in plants: An integrated view on physiology and biochemistry. Environ. Exp. Bot..

[6] Rogo U., Viviani A., Pugliesi C., Fambrini M., Usai G., Castellacci M., Simoni S. (2025). Improving Crop Tolerance to Abiotic Stress for Sustainable Agriculture: Progress in Manipulating Ascorbic Acid Metabolism via Genome Editing. Sustainability.

[7] Yang G., Li H., Xin Y., Yu H., Chen L., Li L., Han D. (2020). Effect Abscisic Acid on Expression of its Synthesis Key Enzyme Gene RiNCED1 and RiCYP707A1 and Quality of Raspberry (*Rubus idaeus*) Fruits. Int. J. Agric. Biol..

[8] Shu P., Zhang Z., Wu Y., Chen Y., Li K., Deng H., Zhang J., Zhang X., Wang J., Liu Z. (2023). A comprehensive metabolic map reveals major quality regulations in red-flesh kiwifruit (*Actinidia chinensis*). New Phytol..

[9] Yang H., Zhang X., Wu R., Tang X., Yang Y., Fan X., Gong H., Grierson D., Yin X., Li J. (2024). Integrated metabolomic and transcriptomic analyses provide comprehensive new insights into the mechanism of chitosan delay of kiwifruit postharvest ripening. Postharvest Biol. Technol..

[10] Du M., Sun C., Deng L., Zhou M., Li J., Du Y., Ye Z., Huang S., Li T., Yu J. (2025). Molecular breeding of tomato: Advances and challenges. J. Integr. Plant Biol..

[11] Nie H., Yang X., Zheng S., Hou L. (2024). Gene-Based Developments in Improving Quality of Tomato: Focus on Firmness, Shelf Life, and Pre-and Post-Harvest Stress Adaptations. Horticulturae.

[12] Gómez-García M.D., Ochoa-Alejo N. (2016). Predominant role of the l-galactose pathway in l-ascorbic acid biosynthesis in fruits and leaves of the *Capsicum annuum* L. chili pepper. Braz. J. Bot..

[13] Hasanuzzaman M., Bhuyan M.B., Zulfiqar F., Raza A., Mohsin S.M., Al Mahmud J., Fujita M., Fotopoulos V. (2020). Reactive Oxygen Species and Antioxidant Defense in Plants under Abiotic Stress: Revisiting the Crucial Role of a Universal Defense Regulator. Antioxidants.

[14] Cárcamo-Fincheira P., Nunes-Nesi A., Soto-Cerda B., Inostroza-Blancheteau C., Reyes-Díaz M. (2024). Ascorbic acid metabolism: New knowledge on mitigation of aluminum stress in plants. Plant Physiol. Biochem. PPB.

[15] Liu S., Yu L., Liu L., Yang A., Huang X., Zhu A., Zhou H. (2023). Effects of ultraviolet-B radiation on the regulation of ascorbic acid accumulation and metabolism in lettuce. Horticulturae.

[16] Zhang Y.Y., Li H.X., Shu W.B., Zhang C.J., Ye Z.B. (2011). RNA interference of a mitochondrial APX gene improves vitamin C accumulation in tomato fruit. Sci. Hortic..

[17] Li H., Liu J.-X., Wang Y., Zhuang J. (2020). The ascorbate peroxidase 1 regulates ascorbic acid metabolism in fresh-cut leaves of tea plant during postharvest storage under light/dark conditions. Plant Sci..

[18] Liang Z., Xu H., Qi H., Fei Y., Cui J. (2024). Genome-wide identification and analysis of ascorbate peroxidase (APX) gene family in hemp (*Cannabis sativa* L.) under various abiotic stresses. PeerJ.

[19] González-Gordo S., Rodríguez-Ruiz M., López-Jaramillo J., Muñoz-Vargas M.A., Palma J.M., Corpas F.J. (2022). Nitric oxide (NO) differentially modulates the ascorbate peroxidase (APX) isozymes of sweet pepper (*Capsicum annuum* L.) fruits. Antioxidants.

[20] Bulley S., Wright M., Rommens C., Yan H., Rassam M., Lin-Wang K., Andre C., Brewster D., Karunairetnam S., Allan A.C. (2012). Enhancing ascorbate in fruits and tubers through over-expression of the l-galactose pathway gene GDP-_L_-galactose phosphorylase. Plant Biotechnol. J..

[21] Cutignano A., Chiuminatto U., Petruzziello F., Vella F.M., Fontana A. (2010). UPLC–MS/MS method for analysis of sphingosine 1-phosphate in biological samples. Prostaglandins Other Lipid Mediat..

[22] Balcke G.U., Handrick V., Bergau N., Fichtner M., Henning A., Stellmach H., Tissier A., Hause B., Frolov A. (2012). An UPLC-MS/MS method for highly sensitive high-throughput analysis of phytohormones in plant tissues. Plant Methods.

[23] Li Y., Tian Y., Zhou X., Guo X., Ya H., Li S., Yu X., Yuan C., Gao K. (2024). Widely targeted metabolomics reveals differences in metabolites of *Paeonia lactiflora* cultivars. PLoS ONE.

[24] Chai G., Qi G., Cao Y., Wang Z., Yu L., Tang X., Yu Y., Wang D., Kong Y., Zhou G. (2014). Poplar PdC3H17 and PdC3H18 are direct targets of PdMYB3 and PdMYB21, and positively regulate secondary wall formation in Arabidopsis and poplar. New Phytol..

[25] Lachmann A., Clarke D.J., Torre D., Xie Z., Ma’Ayan A. (2020). Interoperable RNA-Seq analysis in the cloud. Biochim. Biophys. Acta (BBA)-Gene Regul. Mech..

[26] Anders S., Huber W. (2010). Differential expression analysis for sequence count data. Genome Biol..

[27] Kanehisa M., Furumichi M., Sato Y., Ishiguro-Watanabe M., Tanabe M. (2020). KEGG: Integrating viruses and cellular organisms. Nucleic Acids Res..

[28] Alberts A., Moldoveanu E.-T., Niculescu A.-G., Grumezescu A.M. (2025). Vitamin C: A Comprehensive Review of Its Role in Health, Disease Prevention, and Therapeutic Potential. Molecules.

[29] Mellidou I., Kanellis A.K. (2023). Deep inside the genetic regulation of ascorbic acid during fruit ripening and postharvest storage. Postharvest Biol. Technol..

[30] Chiaiese P., Corrado G., Minutolo M., Barone A., Errico A. (2019). Transcriptional Regulation of Ascorbic Acid During Fruit Ripening in Pepper (*Capsicum annuum*) Varieties with Low and High Antioxidants Content. Plants.

[31] Castro J.C., Castro C.G., Cobos M. (2023). Genetic and biochemical strategies for regulation of L-ascorbic acid biosynthesis in plants through the L-galactose pathway. Front. Plant Sci..

[32] Yuan J., Sun B., Shen C., Chen R., Zhang Y., Xu Y., Li S., Guo X. (2024). Functional analysis of GDH from Chinese cabbage (BrGDH) involved in ascorbic acid synthesis and response to methyl jasmonate. Sci. Hortic..

[33] Tahir H., Sajjad M., Qian M., Haq M.Z.U., Tahir A., Chen T., Shaopu S., Farooq M.A., Ling W., Zhou K. (2024). Transcriptomic analysis reveals dynamic changes in glutathione and ascorbic acid content in Mango pulp across growth and development stages. Horticulturae.

[34] Dowdle J., Ishikawa T., Gatzek S., Rolinski S., Smirnoff N. (2007). Two genes in Arabidopsis thaliana encoding GDP-L-galactose phosphorylase are required for ascorbate biosynthesis and seedling viability. Plant J..

[35] Zhang Y., Peng Y., Zhang H., Gao Q., Song F., Cui X., Mo F. (2024). Genome-Wide Identification of APX Gene Family in Citrus maxima and Expression Analysis at Different Postharvest Preservation Times. Genes.

[36] Tüfekçi E.D., Tellioğlu B., Aygören A.S., Yaprak E., Ilhan E. (2025). Genome-wide characterization of ascorbate peroxidase (APX) gene family in *Phaseolus vulgaris* L. of response to multiple abiotic stresses. S. Afr. J. Bot..

[37] Wang Y.J., Wisniewski M., Meilan R., Cui M.G., Webb R., Fuchigami L. (2005). Overexpression of cytosolic APX1 in Arabidopsis confers enhanced tolerance to oxidative stress. Plant Physiol. Biochem..

